# Willingness to Be Vaccinated Against COVID-19 Among People With HIV in the United States: Results From a National Survey

**DOI:** 10.3389/fmed.2022.886936

**Published:** 2022-06-30

**Authors:** Jeffrey A. Wickersham, Jaimie P. Meyer, Sheela Shenoi, Frederick L. Altice, Lydia Aoun Barakat, Michael Virata, Miriam Olivares, Francesca Maviglia, Antoine Khati, Roman Shrestha

**Affiliations:** ^1^Department of Internal Medicine, Section of Infectious Diseases, AIDS Program, Yale School of Medicine, New Haven, CT, United States; ^2^Department of Chronic Disease Epidemiology, Yale School of Public Health, New Haven, CT, United States; ^3^Department of Epidemiology of Microbial Diseases, Yale School of Public Health, New Haven, CT, United States; ^4^Marx Science and Social Science Library, Yale University Library, Yale University, New Haven, CT, United States; ^5^Department of Allied Health Sciences, University of Connecticut, Storrs, CT, United States; ^6^Institute for Collaboration on Health, Intervention, and Policy, University of Connecticut, Storrs, CT, United States

**Keywords:** COVID-19, HIV, people with HIV, vaccine hesitancy, vaccine uptake

## Abstract

**Background:**

Approximately 215 million Americans have been fully vaccinated for COVID-19, representing over 65% of the total population. People with HIV (PWH) may be more susceptible to COVID-19 infection or severe disease, elevating the importance of COVID-19 vaccination uptake in the population. We report results from a national survey of PWH to evaluate the likelihood of receiving a COVID-19 vaccine.

**Methods:**

We conducted an online survey of 1,030 PWH living in the United States between December 6, 2020 and January 8, 2021 to evaluate likelihood of receiving a COVID-19 vaccine.

**Results:**

Overall, participants were highly willing to be vaccinated, with 83.8% stating they “strongly agree” (65.7%) or “somewhat agree” (18.1%). Participants' top vaccine-related concerns were side-effects (39.3%), safety (14.7%), and fair/equitable distribution of the vaccine to affected communities (13.6%). Participants were more willing to be vaccinated if they reported receiving an annual influenza vaccination (*p* < 0.001), had previously tested positive for (*p* = 0.043) COVID-19, had been hospitalized for (*p* = 0.027) COVID-19 infection, or had an undetectable HIV viral load (*p* = 0.002). Black (*p* < 0.001), politically conservative (*p* < 0.001), and participants with an annual income of ≤ $19,999 (*p* = 0.005) were significantly less willing to be vaccinated for COVID-19.

**Conclusions:**

The vast majority of PWH were willing to be vaccinated, though predominantly those who were already engaged in HIV care or directly affected by COVID-19. Findings from this large survey of PWH suggest intensive outreach efforts are needed to support engagement in vaccination programs, particularly among Black and politically conservative PWH.

## Introduction

High uptake of COVID-19 vaccination is crucial to achieving the level of immunization coverage needed to end the global COVID-19 pandemic (1–3). Prioritizing vaccines for those populations most vulnerable to the disease, including persons who are immunocompromised, has been a key strategy for reducing COVID-19 hospitalizations and in-hospital mortality. Recent evidence suggests that people with HIV (PWH) are at increased risk of hospitalization and death from COVID-19 (4, 5). Data from over 15,000 cases of COVID-19 among PWH showed they were 13% more likely to be hospitalized and had a 30% greater risk of death from COVID-19, regardless of age, sex, disease severity at presentation, and co-morbidities (4). In response, the World Health Organization issued a report, urging that PWH have continued access to antiretroviral treatment during the pandemic and receive priority access to COVID-19 vaccination (6).

However, several challenges persist in ensuring high coverage of COVID-19 vaccination among PWH. A study of COVID-19 vaccine uptake among PWH in Oregon found that as of June 2021 only about two-thirds of PWH had received the vaccine; younger PWH, Hispanic/Latinx PWH, and PWH who inject drugs or reside in rural areas had lower vaccine uptake (7). Attitudes toward vaccination also play a role in vaccine uptake. To date, only two published studies have examined attitudes toward COVID-19 vaccination among PWH in the United States; however, they focus on specific subpopulations (e.g., racial-ethnic minorities), with relatively small sample sizes (8, 9). Understanding attitudes toward COVID-19 vaccination among a broad cross-section of PWH can inform larger-scale interventions to improve vaccine uptake and guide vaccine implementation strategies and programs. In this study, we report findings from a national survey of PWH in the United States regarding their willingness to be vaccinated against COVID-19.

## Methods

We conducted a cross-sectional online survey of PWH living in the United States (*N* = 1,030) between December 2020 and January 2021 to evaluate their willingness to be vaccinated against COVID-19 (10). Participants were recruited through targeted social media advertising to participate in an online survey. Participation was limited to adults 18 years or older with self-reported HIV infection. To control for potential duplicate entries, we followed published procedures to ensure data integrity in internet-based research (11). Participation in this study took approximately 10 min. As compensation, participants had the opportunity to take part in a raffle to win 1 of 5 $100 gift cards. Completion of the survey was not required to enter the raffle. The study was approved by the Yale University institutional review board.

Willingness to be vaccinated against COVID-19 was measured with a single-item question: “*When a vaccine for COVID-19 becomes available, I will get it*.” Participants responded using a 5-point Likert scale (from 1 = strongly disagree to 5 = strongly agree), consistent with other measures of COVID-19 vaccine willingness among the general adult population assessed ordinally and dichotomized for analysis (12). Participants were then asked what would make them more likely to get the COVID-19 vaccines and were able to select all options that applied to them. The survey also collected information about participants' socio-demographic characteristics (e.g., age, sex, sexual orientation, race, education level, and income level), political orientation (e.g., conservative, liberal), HIV & health-related attributes (e.g., time living with HIV, CD4 count, HIV viral load, receipt of annual influenza vaccine) and COVID-19 history and experiences (e.g., prior COVID-19 testing history, prior COVID-19 diagnosis).

We used multivariable logistic regression to explore the association of willingness to be vaccinated against COVID-19 with selected covariates. Candidate covariates were selected based on previous literature on vaccine hesitancy (9, 10, 13, 14). The reference group for each variable included all participants not belonging to the indicated category (e.g., the reference group for “Black” included all participants who did not select “Black” as their race). Statistical significance was set at *p* < 0.05, and all analyses were performed using IBM SPSS 25.

## Results

Participants were mostly male (89.7%), White/Caucasian (66.0%), and gay or lesbian (84.5%) (13). Participants' mean age was 50.7 years (SD = 12.5), and the mean time living with HIV was 17.0 years (SD = 11.1). Overall, participants were highly willing to be vaccinated, with 83.8% stating they “strongly agree” (65.7%) or “somewhat agree” (18.1%) to receive a COVID-19 vaccine when available.

In the multivariable logistic regression model (Table 1), participants who were Black (aOR = 0.47, *p* = 0.008), politically conservative (aOR = 0.39, *p* = 0.002), or had an annual income of ≤$19,999 (aOR = 0.55, *p* = 0.005) were significantly less willing to receive a COVID-19 vaccine, whereas participants who reported being vaccinated annually for influenza (aOR = 6.01, *p* < 0.001) or identified as politically liberal (aOR = 2.63, *p* < 0.001) were more willing to be vaccinated, after adjusting for socio-demographic characteristics, political orientation, HIV & health-related attributes, and COVID-19 history and experiences.

**Table 1:** Bivariate and multivariable logistic regression of COVID-19 vaccine willingness (*N* = 1,030).

**Variables**	**Total Sample**	**OR**	**95%CI**	***p***	**aOR**	**95%**	***p***
**Socio-demographic**							
Male sex	924 (89.7)	3.28	2.11–5.08	<0.001	1.42	0.71–2.87	0.320^*^
Race: Black	116 (11.3)	0.25	0.16–0.38	<0.001	0.47	0.27–0.83	0.008^*^
Race: White	680 (66.0)	2.21	1.57–3.08	<0.001	1.14	0.72–1.80	0.572^*^
Median age (years)	53	1.02	1.01–1.03	0.002	1.01	0.98–1.02	0.507^*^
Education: bachelor's or higher	507 (49.2)	2.31	1.62–3.28	<0.001	1.23	0.80–1.88	0.339^*^
Annual income < $19,999	252 (24.5)	0.38	0.27–0.54	<0.001	0.55	0.36–0.84	0.005^*^
Sexual orientation: gay or lesbian	870 (84.5)	3.16	2.15–4.64	<0.001	1.23	0.68–2.23	0.490^*^
**Political orientation**							
Conservative	72 (7.0)	0.23	0.14–0.37	<0.001	0.39	0.21–0.71	0.002^*^
Liberal	679 (65.9)	4.12	2.91–5.82	<0.001	2.63	1.73–4.02	<0.001^*^
**HIV and Health-related attributes**							
Median time living with HIV (years)	17	1.01	0.99–1.02	0.122			
CD4 >200 cells	804 (78.1)	0.63	0.40–0.99	0.045	1.29	0.76–2.20	0.346
HIV Viral Load Undetectable	984 (95.5)	2.65	1.40–5.02	0.003	1.12	0.47–2.66	0.804
Receive annual flu vaccine	867 (84.2)	6.19	4.25–9.02	<0.001	6.01	3.91–9.22	<0.001^*^
**COVID-19 history and experiences**							
Ever been tested for COVID-19	675 (65.5)	1.38	0.98–1.94	0.064			
Ever tested positive for COVID-19	81 (7.9)	0.71	0.40–1.23	0.227			

*OR, odds ratio; aOR, adjusted odds ratio*.

* *statistical significance at p < 0.05*.

**Figure 1:**
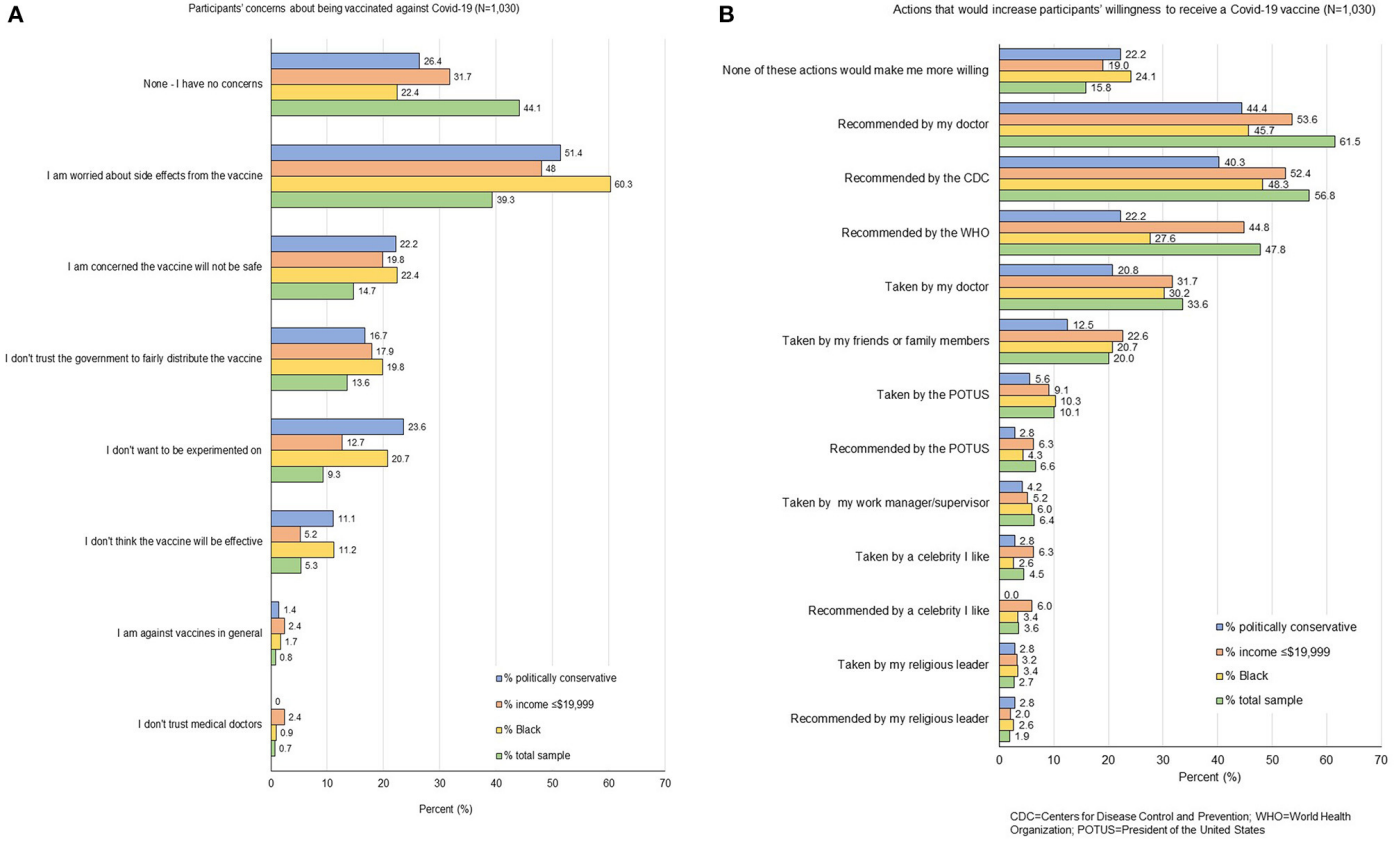
**(A)** Participants' concerns about being vaccinated against COVID-19 (*N* = 1,030). **(B)** Actions that would increase participants' willingness to receive a COVID-19 vaccine (*N* = 1,030).

Participants' primary vaccine-related concerns (Figure 1A) were side-effects (39.3%), safety (14.7%), and fair/equitable distribution of the vaccine to affected communities (13.6%). Side-effects (48.0%), safety (19.8%), and fair/equitable distribution (17.9%) of the vaccine were also the primary concerns among low-income PWH. Among Black PWH and politically conservative PWH, the most commonly reported concerns were side-effects (respectively, 60.3 and 51.4%), safety (22.4 and 22.2%), and not wanting to be experimented on (20.7 and 23.6%).

Participants reported that they would be more willing to get a COVID-19 vaccine if it were recommended by their doctor (61.5%), the Centers for Disease Control and Prevention (CDC; 56.8%), the World Health Organization (WHO; 47.8%), or if their doctor reported having been vaccinated (33.6%; Figure 1B). These priorities were similar among politically conservative PWH and low-income PWH. Among Black PWH, however, a recommendation by the CDC was the action most endorsed to increase vaccine willingness (48.3%), followed by a recommendation (45.7%) or a report of having been vaccinated (30.2%) by their doctor, and a recommendation by the WHO (27.6%).

Black PWH and conservative PWH appeared somewhat less likely to increase their willingness to be vaccinated than low-income PWH, with 24.1% of Black PWH and 22.2% of conservative PWH declaring that none of the proposed actions would make them more willing to get vaccinated, compared to 19% of low-income PWH. A recommendation by the WHO would increase the willingness of almost half (44.8%) of low-income PWH, but only 27.6% of Black PWH and 22.2% of conservative PWH. A recommendation by a doctor was also slightly more influential among low-income PWH (53.6%) than among Black or conservative PWH (45.7 and 44.4%, respectively). A CDC recommendation was more influential among Black and low-income PWH (48.3 and 52.4%, respectively) than among conservative PWH (40.3%). Similarly, 30.2% Black PWH and 31.7% low-income PWH reported being more willing to be vaccinated if it were recommended by their doctor, compared to only 20.8% of conservative PWH.

## Discussion

Achieving an end to the COVID-19 pandemic hinges on the successful vaccination of a majority of the population. In this national sample of PWH, we found a high degree of willingness to be vaccinated for COVID-19—comparably higher than that of the general U.S. adult population (69%). Side effects and safety were the main vaccine-related concerns among participants. Not wanting to be experimented on was a greater concern among Black and politically conservative PWH, while PWH with an annual income of ≤$19,999 were more concerned about fair and equitable distribution of the vaccine. Importantly, none of the HIV-related variables contributed significantly to COVID-19 vaccine willingness, including participants' CD4 level, HIV viral load status, or time living with HIV.

Our results also reflect current societal race and political divisions. For example, Black PWH who are disproportionately affected by COVID-19 infection and mortality, are also less willing to be vaccinated, further exacerbating race-based disparities. Medical mistrust stemming from histories and experiences of medical abuse and racism within the healthcare system affects Black individuals' access to care in the U.S. (15), and has been found to negatively impact willingness to receive the COVID-19 vaccine among Black PWH (9). Similar to politically conservative US residents at large (16), conservative PWH were also less likely to be willing to receive a COVID-19 vaccine, which may be attributed to the politicization of the COVID-19 pandemic.

Our study presents some limitations. The social media-based recruitment strategy means that this self-selecting sample may have been affected by some selection bias. Indeed, compared to overall PWH in the US, White/Caucasian individuals were overrepresented in this sample (17), affecting the generalizability of results. Additionally, the survey was conducted before the COVID-19 vaccine was widely available. Therefore, willingness to be vaccinated and concerns about the vaccine may have changed now that a significant portion of the US population has received at least one dose.

Despite these limitations, this is the first study to our knowledge to examine attitudes toward the COVID-19 vaccine in a large, national sample capturing a broad cross-section of PWH in the U.S. These results offer a first step toward identifying those PWH most likely to decline vaccination and inform the development of effective, targeted health communication to reduce COVID-19 vaccine refusal among different demographic and social groups.

While our findings identify alarming challenges, they also present an opportunity to combat COVID-19. A recommendation by their doctor or the CDC would increase many PWH's willingness to be vaccinated. Clear and accessible information about the process of development of the COVID-19 vaccine and a frank discussion about safety issues and side effects with a trusted healthcare provider may help alleviate some PWH's concerns about the vaccine. Conducting intensive tailored community outreach efforts, identifying trusted sources of information, closing gaps in health equity, and engaging formal and informal opinion leaders within the HIV community will be critical to supporting engagement in vaccination programs.

## Data Availability Statement

The raw data supporting the conclusions of this article will be made available by the authors, without undue reservation.

## Ethics Statement

The studies involving human participants were reviewed and approved by Institutional Review Board, Yale University. Written informed consent for participation was not required for this study in accordance with the national legislation and the institutional requirements.

## Author Contributions

This manuscript was initially conceptualized and written by JW and RS. All authors meet the criteria for authorship, have made substantial contributions to various facets of the manuscript, reviewed, edited, contributed significantly to writing subsequent versions of the manuscript, read, and approved the final manuscript.

## Funding

This work was supported by grants from Yale School of Medicine (COVID-19 Research Funding), the National Institute on Drug Abuse (K01 DA051346 for RS), and career development funding (for JM) was provided by Doris Duke Charitable Foundation.

## Conflict of Interest

The authors declare that the research was conducted in the absence of any commercial or financial relationships that could be construed as a potential conflict of interest.

## Publisher's Note

All claims expressed in this article are solely those of the authors and do not necessarily represent those of their affiliated organizations, or those of the publisher, the editors and the reviewers. Any product that may be evaluated in this article, or claim that may be made by its manufacturer, is not guaranteed or endorsed by the publisher.
